# Screening program of school-going children

**DOI:** 10.4103/0301-4738.64127

**Published:** 2010

**Authors:** Sourabh Aggarwal

**Affiliations:** University College of Medical Sciences, New Delhi, India

Dear Editor,

I read the article 'Effectiveness of using teachers to screen eyes of school-going children in Satna district of Madhya Pradesh, India'[1] with due interest. The authors have done a commendable job for undertaking such a study. It is quite important to review the health programs initiated so that lacunae can be identified and the effectiveness of such programs improved. I would like to add my views on the issue.

The children not attending school can be covered comprehensively by promoting regular eye camps with the aid of the local Government and encouraging the people to attend the same by proper use of media. The high false-positive rate in the study,[1] adding to the burden of the ophthalmic assistant is worrisome. It can be reduced by short-term periodic training of concerned teachers to upgrade their knowledge. But more worrisome is the false-negative rate[1] which though less is of serious concern since these children fail to make use of healthcare when they are in need. The teachers should have a high index of suspicion and should refer the children whenever they are in doubt. This initiative, inspite of adding a little burden to the healthcare clinics, will significantly reduce the morbidity and will contribute to the long-term health of the children.
